# Workaholism as a Mediator between Work-Related Stressors and Health Outcomes

**DOI:** 10.3390/ijerph15010073

**Published:** 2018-01-05

**Authors:** Cecilie Schou Andreassen, Ståle Pallesen, Torbjørn Torsheim

**Affiliations:** 1 Department of Clinical Psychology, Faculty of Psychology, University of Bergen, 5020 Bergen, Norway; 2 Department of Psychosocial Science, Faculty of Psychology, University of Bergen, 5020 Bergen, Norway; Staale.Pallesen@uib.no (S.P.); Torbjoern.Torsheim@uib.no (T.T.)

**Keywords:** workaholism, job demand–control–social support, effort–reward imbalance, burnout, insomnia, general health

## Abstract

It is currently unknown if unfavorable working conditions, reflected by the demand–control–support model and the effort–reward imbalance model, directly influence health or if the effects may be mediated by work-related attitudes and behaviors such as workaholism. In the present study, 988 employees (55.6% males, mean age 36.09, SD = 9.23) from a large consultant firm participated in a cross-sectional survey assessing work variables such as job demands, job control, social support, effort, reward, and overcommitment. Workaholism was also assessed together with eight different health-related outcomes. Although direct effects of the work stressors on health were found on most health outcomes, the work-related stressors were overall strongly related to workaholism (R^2^ = 0.522), which, in turn, was positively related to four (anxiety/insomnia, somatic symptoms, emotional exhaustion, and social dysfunction) of the eight outcome variables. Of a total of 40 relationships between work-related stressors and health outcomes, workaholism fully mediated three of these, and partly mediated 12. Overall, the study suggests that the effects of work-related stressors on health in many cases may be mediated by workaholism.

## 1. Introduction

Workaholism can be defined as “being overly concerned about work, to be driven by strong and uncontrollable work motivation, and to spend so much energy and effort into work that it impairs private relationships, spare-time activities and/or health” [1] (p. 8). In line with this definition, workaholism has been related to a vast array of negative outcomes, such as impaired health [2], low job and life satisfaction [3], work–family conflicts [4], sleep problems [5], exhaustion [6], as well as impaired job performance [7], and sickness absence [8].

Regarding the antecedents of workaholism, one can on a general basis differentiate between individual and situational antecedents. Individual antecedents typically encompass various personality factors (e.g., neuroticism, narcissism, perfectionism, and Type A behavioral pattern) [9,10,11,12], upbringing and family factors [13,14], as well as demographic variables [15].

In terms of situational antecedents much emphasis has been put on the effects of different organizational stressors. One of the most cited and prevailing models in this realm is the job demand–control (JDC) model [16]. High job demands are regarded as straining, and often force workers to deal with a lot of work and to work at a high pace, and are consequently assumed to impair employee health. Control over the work situation is assumed to have an opposite effect on health, either directly (additive component), or as a buffer (interactive component) [16]. Studies have shown that the JDC-model can predict outcomes, for example in terms of cardiovascular events [17], autonomic nervous system activity [18], neck and/or shoulder disorders [19], and common mental disorders [20]. Later, the model has been expanded by adding social support at work as a third component, resulting in the job demand–control–support (JDCS) model [21]. Accordingly, the highest risk of ill-health is to be expected among those experiencing high job demands, low job control, and low social support at work [22]. Empirical studies also attest to the predictions of the JDCS-model [23].

Research has consistently shown positive associations between job demands and workaholism [6,24,25,26,27,28,29]. The relationship between job control and workaholism seems more ambiguous, as three previous studies found no relation [7,29,30], one study reported a negative relation [26], and one reported a positive association between job control and workaholism [28]. A study looking into how demand and control interacted in terms effects of workaholism reported (at odds with what was hypothesized) that workaholism increased more strongly with increasing demands for workers with high, compared to those with low, job control [28]. Also, results from studies on the relationship between social support from colleagues and workaholism appear equivocal. Two studies reported a negative relationship between workaholism and indicators of social support [26,29], whereas two studies found no relationship between these constructs [24,27].

Another model of organizational stress is the effort–reward imbalance (ERI) model [31,32]. This model assumes that effort at work is spent as part of a contract, based on the norm of social reciprocity, in such a way that a symmetry between effort and reward (e.g., salary, esteem, career opportunities and job security) is expected. Asymmetry (e.g., high effort and low reward) is assumed to cause sustained strain reactions. Some workers are assumed to have a high need for approval. Those workers typically expose themselves to high demands at work, which, in turn, is assumed to make them especially at risk of strain from non-symmetric exchange [31,32]. Several studies attest to the negative effects of ERI, e.g., in terms of depressive reactions [33], coronary heart disease [34], and lowered immunity [35]. We have so far not been able to identify studies investigating the relationship between the ERI-model and workaholism.

As it is conceivable that work-related stressors can cause workaholism, and since workaholism consistently is related to a vast array of poor outcomes, we decided to investigate whether the JDCS-model and the ERI-model could predict workaholism, and if workaholism mediates the effects of these two models on health outcomes (burnout, insomnia, and mental health), and to which degree the two work-related stressor models directly influence health outcomes.

## 2. Materials and Methods

### 2.1. Procedure and Participants

The present study is based on a survey study conducted among employees in a large international consultant firm. An invitation to participate in a survey about working environment was sent on email to about 1500 employees working in Norway. The employees completed the survey online and received automatic feedback on the scores on some of the instruments. It was voluntary to take part in the survey, and the data collection was anonymous. The project was approved by the Regional Committee for Medical and Health Research Ethics, region West (2011/1629). In total, 988 employees did participate (yielding a response of about 66%), whereof 44.4% were females and 55.6% were males. The mean age of the sample was 36.09 (SD = 9.23) years. The majority of the participants were married/cohabitating with partner (81.0%), were living without children (51.3%), and held a master degree (75.5%) as highest completed education. In terms of professional position, 4.7% were administrative staff, 18.3% were associates, 27.6% were senior associates, 5.9% were supervisors, 16.1% were managers, 10.1% were senior managers, 7.9% were directors, 8.3% were partners, and 1.1% reported “other” positions.

### 2.2. Variables

The participants provided information about sex, age, marital status, number of children in the household, as well as level of education. In addition, the following instruments were administered:

- Swedish Demand–Control–Support Questionnaire (DCSQ). This instrument assesses three dimensions of the working environment. Demand is assessed with five items (e.g., “Does your job require you to work very fast?”), decision latitude/control is assessed with five items (e.g., “Do you have the opportunity to learn new things in your work?”), and social support is assessed with six items (e.g., “There is good collegiality at work”). All items are answered on a four-point scale (1–4). High scores reflect high levels of job demand, job control, and social support at work, respectively [36]. The Cronbach’s alphas for the demand, control, and social support subscales in the present study were 0.70, 0.61, and 0.83, respectively.
- Effort–Reward Imbalance Questionnaire (ERIQ). The ERIQ contains 22 items assessing effort (5 items; e.g., “I have a lot of responsibility in my job”), reward (11 items; e.g., “I receive the respect I deserve from my superiors”), and overcommitment (6 items; e.g., “I get easily overwhelmed by time pressures at work”). The effort items are answered on a five-point Likert scale ranging from ‘disagree’ (1) to ‘agree, and I am very distressed’ (5), the reward items are answered on a five-point Likert scale ranging from ‘disagree, and I am very distressed’ (1) to ‘agree’ (5), whereas the overcommitment items are answered on a four-point Likert scale ranging from strongly ‘disagree’ (1) to ‘strongly agree’ (4). A ratio reflecting balance between effort and reward is computed according to a formula (effort/reward × 0.4545) where the lower the score the better. Values above 1.0 indicate a high amount of effort spent that is not met by the rewards received or expected [31]. The alphas for the effort, reward, and overcommitment scales in the present study were 0.72, 0.82, and 0.75, respectively.
- Bergen Work Addiction Scale (BWAS). Workaholism was assessed by the BWAS comprising seven items, all reflecting general addiction criteria (i.e., salience, tolerance, mood modification, relapse, withdrawal, conflict, and problems) experienced during the past year (e.g., “How often the last year have you become stressed if you have been prohibited from working?”). Each item is answered on a five-point Likert scale ranging from ‘never’ (1) to ‘always’ (5). Higher scores reflect higher levels of workaholism [37]. The Cronbach’s alpha of the BWAS in the present study was 0.81.
- Bergen Insomnia Scale (BIS). The BIS consists of six items designed to measure insomnia (e.g., “During the past month, how many days a week has it taken you more than 30 min to fall asleep after the light was switched off?”). Each item is scored from 0 to 7, according to the number of days per week the last month that the given problem has been experienced. A composite score is calculated by adding the scores of the individual items [38]. In the present study, the alpha of the BIS was 0.81.
- Maslach Burnout Inventory-General Survey (MBI-GS). The MBI-GS contains 16 items, assessing three dimensions related to burnout. One dimension is reflected by the emotional exhaustion scale (5 items), which assesses feelings of physical and emotional resource depletion. The second dimension is cynicism (5 items), which reflects issues such as depersonalization (distant attitude towards one’s job); and the third is professional efficacy (6 items), which is based on items assessing personal accomplishment at work. All items are answered on a seven-point scale, ranging from ‘never during the last year’ (0) to ‘daily’ (6) [39]. The alphas of the emotional exhaustion, cynicism, and professional efficacy subscales in the present study were 0.86, 0.80, and 0.85, respectively.
- General Health Questionnaire-28 (GHQ-28). The GHQ-28 is a measure of general health problems and consists of four dimensions, each reflected by seven items: Somatic symptoms (e.g., “Felt that you are ill”), anxiety/insomnia (e.g., “Been feeling nervous and strung-up all the time”), social dysfunction (e.g., “Felt that you are playing a useful part in things”), and severe depression (e.g., “Felt that life is entirely hopeless”). Each item is rated on a four-point Likert scale (1–4; e.g., ‘better/healthier than usual’ to ‘much more/worse than usual’), where higher scores reflect more problems [40,41]. The alphas of the somatic symptom, anxiety/insomnia, social dysfunction, and severe depression subscales in the present study were 0.82, 0.85, 0.83, and 0.83, respectively.

### 2.3. Statistical Analyses

Descriptive statistics were calculated in terms of percentages, means, and standard variation. Zero-order relationships between the variables in the model were expressed in terms of Pearson product–moment correlation coefficients. Mediation models were tested using Mplus 8 (Muthén & Muthén, Los Angeles, CA, USA). A path model was specified, where central variables stemming from the JDCS-model (demand, control, and support) and the ERI-model (effort–reward ratio (ER-ratio) and overcommitment) comprised the independent variables (X). Workaholism constituted a mediator (Z) in the model, whereas burnout (emotional exhaustion, cynicism, and professional efficacy), insomnia, and general health (somatic symptoms, anxiety/insomnia, social dysfunction, and severe depression) comprised the dependent variables (Y). Using established terminology, the path model contained three kinds of paths: Paths from the independent variables to the mediator (a paths), paths from the mediator to the dependent variables (b paths), and direct paths from the independent variables to the dependent variables (c’ paths). In the case of *full mediation*, the multiplicative a × b term would be statistically significant, and the c’ paths would not be significant. *Partial mediation* was indicated when the a × b term and the c’ paths were statistically significant. Partial and full mediation were tested through Sobel test of the multiplicative a × b term. Using this framework, the association between independent and dependent variables can be decomposed into direct (non-mediated), indirect (mediated), and total effects. The mediation framework thus included estimates of direct and indirect and total effects. The percentage of missing data on the composite scores of the scales involved ranged from 2.8% to 12.9%. Missing data was estimated with the Mplus full information likelihood procedure, utilizing all available data. As the analyses were run with a bootstrap procedure, and since the sample size was high, no test of normal theory assumptions was needed as the full information likelihood procedure would produce unbiased estimates.

## 3. Results

Descriptive statistics of how the sample scored on the instruments included in the present study are presented in Table 1 (in terms of means and standard deviations).

Table 2 shows the zero-order correlation coefficients between the variables in the model. The correlation coefficients ranged from −0.474 (between social support and cynicism) to 0.649 (between somatic symptoms and anxiety/insomnia).

Table 3 shows the standardized coefficients from the mediation path model. Job demands and job control from the JDCS-model were positively and significantly related to workaholism, whereas social support at work was not. The effort–reward ratio and work overcommitment, stemming from the ERI-model, were also positively and significantly related to workaholism. These variables explained in total 52.2% of the variance in workaholism. Workaholism had significant and positive relationships with anxiety/insomnia, somatic health, emotional exhaustion, and social dysfunction, but not with depression, cynicism, insomnia, and professional efficacy. As shown in Table 4, the tests of indirect effects from the mediation path model (independent variable × workaholism) were significant (results not shown) for four of the outcomes. In all, three relationships were fully mediated by workaholism (job demands × anxiety/insomnia, job demands × social dysfunction, and job demands × emotional exhaustion), whereas 12 relationships were partly mediated by workaholism, and 25 relationships showed either a direct effect only or no significant relationship with health outcomes at all. Taken together, the results indicated that a model where workaholism mediated the effects of the work stressor variables on the four aforementioned outcome variables was consistent with the data. Indirect effects including social support as an independent variable was not statistically significant for any of the outcomes.

## 4. Discussion

Overall, the results showed that job demands were positively associated with workaholism, which is in line with several previous studies [6,24,25,26,27,28,29]. High job demands might foster workaholism as an attempt to escape uncomfortable stress (negative reinforcement), and may also convey descriptive norms promoting excessive work [28,42]. In the present study job control was positively associated with workaholism. Job control may facilitate workaholism in the sense that having influence over what and how work is to be done, without interference from supervision, allows the individual to choose the ‘workaholic way of working’. Alternatively, having the ability to control tasks and work pace, and having freedom from monitoring/supervision, may be highly valued and may motivate the worker to invest heavily in work with a view to upholding, and not losing, this valued freedom [28]. In the present study, social support at work was associated with workaholism in the bivariate analysis, but not in the multivariate analysis. The former finding is in line with other studies [26,29], and may reflect that a socially supportive climate may prevent workaholism from developing in the first place, or diminish the negative consequences of it through helping employees to enjoy their work. Other mechanisms may also be involved, such as social distraction from work and instrumental help so that one does not need to do everything alone. An additional speculation is that some may work obsessively in an environment where social support is lacking, in order to gain support and social recognition. Finally, being addicted to work may also imply that there is little time for interaction with colleagues, thus undermining the potentially positive impact of social support [28]. Still, in the multivariate analysis social support was not related to workaholism, which is in agreement with at least two previous studies [24,27]. The present study is, to the best of our knowledge, the first that have investigated the relationship between the ERI-model and workaholism. A relatively strong positive relationship between ERI (indexed by the effort–reward ratio) and workaholism was found. One interpretation of this is that workers intensify their work effort with the aim to achieve an increase in reward [43], albeit without success. Another interpretation is that high effort–reward ratios generally are characteristic of workaholics, since they are heavy work investors [44]. The other component of the ERI-model, work overcommitment, was also strongly and positively related to workaholism. This has been reported in other studies [45]. Overcommitment reflects a set of attitudes, behaviors, and emotions that mirror excessive striving for approval and appreciation. Accordingly, people who overcommit are exaggerating their efforts beyond levels usually considered appropriate [46]. However, whether work overcommitment leads to workaholism or more reflects an overlapping construct, which previously has been suggested [47], is a matter for further study.

In the present study, workaholism was positively associated with four of the eight health-related outcome measures. The positive association with anxiety/insomnia is in line with studies linking workaholism to neuroticism [48], as well as with studies linking workaholism to poor sleep [5,49]. Workaholism was furthermore positively related to impaired somatic health, which also has been reported previously [26,50]. That finding is in line with the generally established association between workaholism and impaired health [51]. A positive association between workaholism and emotional exhaustion was also found in the present study. This has previously been demonstrated longitudinally [52], where actually a mutual influence was found. This may reflect a vicious circle whereby workaholism naturally is assumed to cause exhaustion, but also where exhaustion may intensify workaholic behavior, for example in order to conserve work ability and the positive reinforcements work may bring, which would be in line with the conservation of resources theory [53].

The cross-section design of the present study prevents, however, further study into the lagged relationship between workaholism and emotional exhaustion. Moreover, workaholism was in the present study positively related to social dysfunction. This has been reported previously [50], and might suggest that workaholism depletes the worker for resources that otherwise could be invested in social relationships [54]. For all the four outcome variables that workaholism was related to, the product-terms from the mediation path model (independent variable × workaholism) were also significant, suggesting that work-related factors such as job demand, control, effort–reward imbalance, and work overcommitment negatively influence health via workaholism. As such, this model suggests that work-related stressors, like work demands and ERI, seem to foster workaholism, which, in turn, may cause impaired health. In all, three relationships were fully mediated by workaholism (demands→anxiety/insomnia, demands→social dysfunction, and demands→emotional exhaustion). All these involved job demands, and may suggest that job demands in particular influence health via workaholism. Furthermore, 12 relationships between work-related stressors and health outcomes were partly mediated by workaholism. The strongest partly mediated effects seemed to be related to the two components from the ERI-model. Overall, the results of the present study suggest that negative health effects of work stressors in several cases may be (fully or partly) mediated by workaholism.

In addition to the mediating effect via workaholism, the results in terms of direct effects were by and large in line with previous research on work-related stressors. In this realm, it should be noted that job demands were positively related to insomnia and cynicism, whereas control and social support were inversely related to anxiety/insomnia, depression, poor somatic health, insomnia, cynicism, emotional exhaustion, and social dysfunction. Control and social support were positively associated with professional efficacy. These findings are in general in line with previous research on the JDCS-model [23]. Some inconsistent findings were, however, noted as demands was inversely related to somatic health and positively related to professional efficacy. When it comes to the ERI-model, all significant findings, both related to the effort–reward ratio and overcommitment, were in line with the model, in as much as these variables were positively related to negative outcomes and inversely related to positive outcomes.

In terms of implications, the results of the present study suggest that reducing job demands may have positive effects in terms of both workaholism and health. This may imply that workaholism may be prevented by decreasing workload and external pressure [55], by for example changing expectations and norms. Interventions that might improve demands entail increased task variety, more personnel, and more time to plan work [56]. Increasing rewards related to esteem/support and financial/status may also have a protective effect against workaholism and impaired health [57]. Counseling strategies that may include stress management and helping workaholics to find work they enjoy or work that they perceive as highly meaningful have also been suggested [58]. A specific counseling goal for compulsive workaholics might be to reduce the extent to which their behavior is perceived as dysfunctional by themselves and by the organization employing them [59]. Furthermore, counseling based on self-validation has also been suggested, where the workaholic learn to validate and value other self-related aspects than work, such as the spiritual, transcultural-existential, social-cultural, familial, and physical self [60].

### Strengths and Limitations

The present study was based on a cross-sectional design; hence nothing can be firmly concluded regarding the directionality between study variables. Further, the cross-sectional design may make the results vulnerable to the common method bias, as all data were based on self-report [61]. Some of the scales had a rather low internal consistency, which typically weakens relationships with other constructs; although it should be noted that scales with few items naturally often results in relatively low alpha values [62]. Although some outcome variables were not significantly related to workaholism at the bivariate level, they were still (for the sake of consistency in the data analytic approach) included in the multivariate model.

In terms of assets of the present study, it is (to the best of the authors knowledge) the first to have linked both the JDCS-model [16,63] and the ERI-model [31,32] to workaholism, and one of very few studies to regard workaholism as a mediator between work-related stressors and health outcomes. Still, the mediator model presented here should be investigated in future studies, preferably using longitudinal designs. It should further be noted that the present study had a large sample size, providing high statistical power and that the response rate was high. The use of well-validated scales is also an asset of the present study. Although we did not specifically control for sex and age in the reported findings, the results were upheld when controlling for sex and age (results not shown).

## 5. Conclusions

Although direct effects of the work stressors on health was found on most health outcomes, the work-related stressors were overall strongly related to workaholism, which, in turn, were positively related to four of the eight outcome variables. Workaholism fully mediated three and partly mediated 12 of the work stressors–health outcome relationships. All in all, the results suggest that workaholism in many cases may mediate the relationship between work-related stressors and health-related outcomes.

## Figures and Tables

**Table 1:** Descriptive data of the instruments included in the study model.

Instrument	Mean	SD	Score Range
Swedish Demand–Control–Support Questionnaire			
Demand	14.69	2.31	7–20
Control (decision latitude)	15.24	2.11	8–20
Social support	19.53	2.72	9–24
Effort–Reward Imbalance Questionnaire			
Effort	11.97	3.92	5–25
Reward	50.41	5.95	19–55
Effort–reward ratio	0.54	0.23	0.20–2.20
Overcommitment	14.68	3.13	6–24
Bergen Work Addiction Scale	18.65	4.55	7–34
Bergen Insomnia Scale	10.59	7.65	0–39
Maslach Burnout Inventory			
Emotional exhaustion	6.67	5.62	0–28
Cynicism	6.70	5.37	0–28
Professional efficacy	27.72	6.09	5–36
General Health Questionnaire-28			
Somatic symptoms	13.52	3.89	7–27
Anxiety/insomnia	12.24	4.05	7–27
Social dysfunction	14.90	2.70	7–26
Severe depression	8.13	2.28	7–27

SD—standard deviation. Score range—the range of scores for each variable in the present study.

**Table 2:** Zero-order correlations (Pearson product–moment coefficients; r) between study variables.

Scale	1	2	3	4	5	6	7	8	9	10	11	12	13
1. Demand													
2. Control	0.022												
3. Social support	−0.282	0.220											
4. Effort–reward ratio	0.531	−0.058	−0.422										
5. Overcommitment	0.442	−0.066	−0.297	0.552									
6. Bergen Work Addiction Scale	0.494	0.032	−0.293	0.581	0.634								
7. Bergen Insomnia Scale	0.301	−0.138	−0.292	0.386	0.469	0.363							
8. Emotional exhaustion	0.383	−0.193	−0.384	0.589	0.538	0.512	0.467						
9. Cynicism	0.294	−0.344	−0.457	0.401	0.316	0.295	0.333	0.560					
10. Professional efficacy	−0.040	0.322	0.313	−0.127	−0.235	−0.104	−0.218	−0.270	−0.288				
11. Somatic symptoms	0.226	−0.166	−0.272	0.408	0.446	0.403	0.469	0.543	0.360	−0.270			
12. Anxiety/insomnia	0.288	−0.128	−0.327	0.477	0.550	0.461	0.528	0.590	0.390	−0.324	0.649		
13. Social dysfunction	0.212	−0.198	−0.285	0.411	0.356	0.329	0.398	0.547	0.412	−0.320	0.582	0.647	
14. Severe depression	0.157	−0.158	−0.258	0.295	0.277	0.225	0.310	0.448	0.389	−0.263	0.399	0.575	0.484

r’s with absolute values less than 0.066 are non-significant; r’s with absolute values between 0.066 and 0.103 are significant at the 0.05 level; r’s with absolute values above 0.103 are significant at the 0.01 level.

**Table 3:** Standardized coefficients from the mediation path model.

Variables	Mediator	Dependent							
Dependent	WA	Anx	Dep	Som	Ins	Cyn	Exh	PEf	SDy
Workaholism		0.139 **	0.029 ns	0.168 ***	0.058 ns	0.053 ns	0.163 ***	0.031 ns	0.104 *
Demand	0.125 ***	−0.049 ns	−0.030 ns	−0.068 *	0.070 *	0.096 **	0.020 ns	0.081 *	−0.045 ns
Control	0.071 **	−0.072 **	−0.112 **	−0.128 ***	−0.093 **	−0.277 **	−0.150 ***	0.252 ***	−0.159 ***
Social support	−0.019 ns	−0.118 **	−0.133 **	−0.076 *	−0.120 **	−0.283 ***	−0.123 ***	0.242 ***	−0.101 **
ER-ratio	0.321 ***	0.112 ***	0.104 ns	0.120 *	0.028 ns	0.119 **	0.252 ***	−0.056 ns	0.174 ***
Overcommitment	0.398 ***	0.382 ***	0.168 **	0.272 ***	0.345 ***	0.071	0.236 ***	−0.233 ***	0.173 ***
*R-squared*	0.522	0.355	0.126	0.258	0.262	0.335	0.432	0.199	0.207

ER—effort–reward; WA—workaholism; Anx—anxiety/insomnia; Dep—depression; Som—somatic symptoms; Ins—insomnia; Cyn—cynicism; Exh—emotional exhaustion; PEf—professional efficacy; SDy—social dysfunction. * *p* < 0.05, ** *p* < 0.01, *** *p* < 0.001, ns—non significant.

**Table 4:** Statistically significant indirect paths (independent * workaholism).

Independent	Dependent	Via Workaholism	SE	t	*p*	sig
Demand	Anxiety/insomnia	0.017	0.007	2.636	0.008	**
Control	Anxiety/insomnia	0.010	0.005	2.171	0.030	*
Effort–reward ratio	Anxiety/insomnia	0.045	0.014	3.249	0.001	**
Overcommitment	Anxiety/insomnia	0.055	0.017	3.303	0.001	**
Demand	Somatic symptoms	0.021	0.007	2.955	0.003	**
Control	Somatic symptoms	0.012	0.005	2.332	0.020	*
Effort–reward ratio	Somatic symptoms	0.054	0.015	3.690	0.001	***
Overcommitment	Somatic symptoms	0.067	0.018	3.726	0.001	***
Demand	Social dysfunction	0.013	0.007	1.983	0.047	*
Effort–reward ratio	Social dysfunction	0.033	0.015	2.225	0.026	*
Overcommitment	Social dysfunction	0.041	0.019	2.210	0.027	*
Demand	Emotional exhaustion	0.020	0.007	2.785	0.005	**
Control	Emotional exhaustion	0.012	0.005	2.453	0.014	*
Effort–reward ratio	Emotional exhaustion	0.052	0.015	3.516	0.001	***
Overcommitment	Emotional exhaustion	0.065	0.018	3.648	0.001	***

SE—standard error, * *p* < 0.05, ** *p* < 0.01, *** *p* < 0.001.
